# IL-33 prevents age-related bone loss and memory impairment by suppression of Th17 response: evidence in a d-galactose–induced aging mouse model

**DOI:** 10.1093/jbmrpl/ziae101

**Published:** 2024-08-02

**Authors:** Saurabh Kumar Kaushal, Parul, Alok Tripathi, Devendra Pratap Singh, Ankita Paul, Kumari Alka, Shubha Shukla, Divya Singh

**Affiliations:** Division of Endocrinology, CSIR-Central Drug Research Institute, Lucknow, Uttar Pradesh 226031, India; Academy of Scientific and Innovative Research (AcSIR), Ghaziabad, Uttar Pradesh 201002, India; Division of Neuroscience and Ageing Biology, CSIR-Central Drug Research Institute, Lucknow, Uttar Pradesh 226031, India; Division of Endocrinology, CSIR-Central Drug Research Institute, Lucknow, Uttar Pradesh 226031, India; Academy of Scientific and Innovative Research (AcSIR), Ghaziabad, Uttar Pradesh 201002, India; Division of Endocrinology, CSIR-Central Drug Research Institute, Lucknow, Uttar Pradesh 226031, India; Academy of Scientific and Innovative Research (AcSIR), Ghaziabad, Uttar Pradesh 201002, India; Division of Endocrinology, CSIR-Central Drug Research Institute, Lucknow, Uttar Pradesh 226031, India; Division of Neuroscience and Ageing Biology, CSIR-Central Drug Research Institute, Lucknow, Uttar Pradesh 226031, India; Academy of Scientific and Innovative Research (AcSIR), Ghaziabad, Uttar Pradesh 201002, India; Division of Neuroscience and Ageing Biology, CSIR-Central Drug Research Institute, Lucknow, Uttar Pradesh 226031, India; Division of Endocrinology, CSIR-Central Drug Research Institute, Lucknow, Uttar Pradesh 226031, India; Academy of Scientific and Innovative Research (AcSIR), Ghaziabad, Uttar Pradesh 201002, India

**Keywords:** aging, inflammation, immune system, oxidative stress, T cells

## Abstract

Cytokines are the primary mediators of age-related disorders. The IL-17/IL-10 axis plays a crucial role in bone destruction and neuro-inflammation. Additionally, a new Th2 cytokine—IL-33—has gained attention for its potential implications in aging-associated conditions. However, the involvement of IL-33 in aging-mediated bone loss and memory impairment remains unclear and needs further investigation. This study reveals the impact of IL-33 on various aspects of the immune system, bone health, and neural functions. To induce senescence, we used d-galactose for its convenience and fewer side effects. The experimental design involved treating 20-week-old C57BL/6J mice with d-galactose subcutaneously for 10 weeks to induce aging-like effects. Thereafter, IL-33 recombinant protein was administered intraperitoneally for 15 days to evaluate its impact on various immune, skeletal, and neural parameters. The results demonstrated that d-galactose–induced aging led to bone loss and compromised osteogenic parameters, accompanied by increased oxidative stress and neurodegeneration in specific brain regions. Behavioral activities were also affected. However, supplementation with IL-33 mitigated these effects, elevating osteogenic parameters and reducing senescence markers in osteoblast cells in an aging mouse model and exerted neuroprotective potential. Notably d-galactose–induced aging was characterized by high bone turnover, reflected by altered serum levels of CTX, PTH, beta-galactosidase, and P1NP. IL-33 treatment attenuated these effects, suggesting its role in regulating bone metabolism. Furthermore, d-galactose–induced aging was associated with increased differentiation of Th17 cells and upregulation of associated markers, such as STAT-3 and ROR-γt, while downregulating Foxp3, which antagonizes Th17 cell differentiation. IL-33 treatment countered these effects by suppressing Th17 cell differentiation and promoting IL-10–producing T-regulatory cells. Overall, these findings provide insights into the potential therapeutic implications of IL-33 in addressing aging-induced bone loss and memory impairment.

## Graphical Abstract

**Graphical Abstract:**
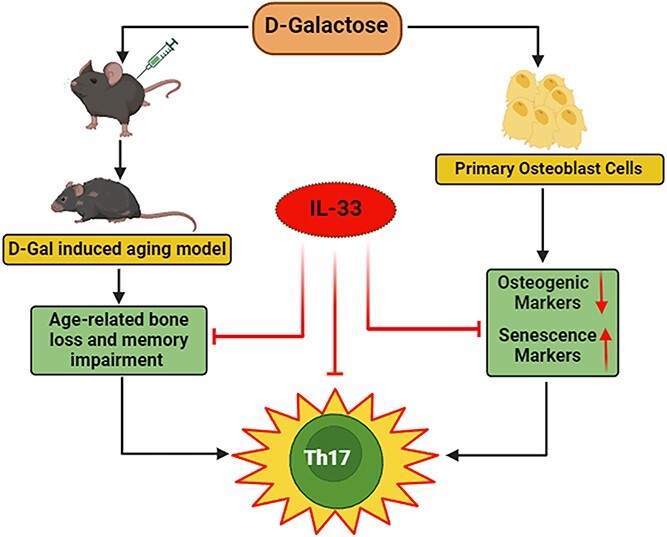

## Introduction

Aging and inflammation are known to be associated with bone loss and memory impairment. The relationship between chronic inflammation, oxidative stress, and the aging process is a complex and multifactorial process.^1^ Many cytokines, like TNF-α and IL-17, have been directly associated with the development of bone-related disorders in both animal models and in patients.^2^ However, the role of the IL-33 cytokine in the pathogenesis of aging-related bone loss and its impact on memory impairment remains unclear. IL-33 cytokine is expressed mainly by stromal cells and is upregulated following proinflammatory stimulation. In addition to its conventional role as a cytokine, IL-33 can also act as an alarmin, a nuclear factor, and a transcription regulator for genes.^3^ It interacts with the receptors ST2 (IL-1RL1) and IL-1 receptor accessory protein (IL-1RAcP) to demonstrate its biological actions. Th2 cells are the primary cells that express these receptors. Whenever cellular harm or injury occurs, IL-33 is discharged into the extracellular space. It acts like an alarm bell for the immune system, signaling that something is wrong and prompting an inflammatory response.^4^ Aberrant signaling by IL-33 is implicated in many diseases, and several studies have postulated targeting the IL-33/ST2 pathway for therapeutic benefits. The protective or destructive role of IL-33 depends on the nature and duration of the disease. IL-33 is highly expressed in normal epithelial cells in many lining tissues, suggesting a role in immune surveillance of tissue damage. IL-33 plays a central role in Treg cell proliferation and function .^5^ IL-33 attenuates sepsis by inhibiting IL-17 receptor signalling.^6^ Although IL-33 is well known for its involvement in inflammatory diseases like asthma and atopic dermatitis, its role in bone and brain metabolism is largely unexplored except for a few sporadic reports.^7^ Ginaldi et al^8^ have shown that IL-33 levels were significantly low and inversely correlated with CTx levels in the serum of postmenopausal, osteoporotic women. Previous studies have indicated that IL-33 inhibits osteoclastogenesis, suggesting a potential role in mitigating osteoporosis.^9^ Fu et al^10^ showed that IL-33 ameliorates Alzheimer’s disease–like pathology, and Alzheimer’s disease is considered to be a risk factor for osteoporosis. Thus, although the background reports indicate a role for IL-33 in the pathogenesis of osteoporosis, the effect of IL-33 exogenous administration on skeletal health has never been explored in an aging osteoporotic animal model. This encouraged us to explore the role of IL-33 in aging bone-loss conditions where the risk of cognitive decline is increased simultaneously. For achieving this goal, we selected the d-galactose (D-gal)–induced accelerated aging mouse model, as there are reports that rats undergoing 6 weeks of D-gal administration to induce aging developed the characteristics of oxidative stress and antioxidants similar to those of 24-month-old, naturally aged rats.^11^d-Galactose instigates aging-inducible oxidative stress in vivo, which is an appropriate model for researching the process of aging-mediated bone loss and memory impairment in mice. This mimics the normal aging process in mice.^12^

## Materials and methods

### Reagents and chemicals

Primers and cell culture materials were procured from Sigma Aldrich (St. Louis, MO). Recombinant mouse IL-33 and the True-Nuclear Mouse Treg Flow Kit were purchased from Biolegend (San Diego, CA, USA). We purchased ionomycin, PMA, and brefeldin-A from Sigma Aldrich (St. Louis, MO, USA). From BD Biosciences (San Diego, CA, USA), FACS tubes and FACS antibodies IL-17A-PE, IL-33 (ST2) BV421, CD4^+^ Allophycocyanin (APC) were obtained. The Hisep used to isolate the PBMCs was purchased from Himedia (Mumbai, India). We procured the cDNA synthesis kit from Invitrogen (Carlsbad, CA, USA). We bought pRB Phospho-Rb, p53, p21, PCNA Proliferating cell nuclear antigen, Runx-2, and type 1 collagen from CST Cell Signaling Technology. Ki-67 and p21 purchased from Affinity. CD4 Imag particles and an Imag cell separation magnet were purchased from BD Biosciences (San Diego, CA, USA).

### Methods

Details on mouse calvarial osteoblast culture, alkaline phosphatase assay, cell viability assay, mineralization assay, GSH measurement, nitrite estimation, lipid peroxidation, behavioral tests, total RNA isolation, quantitative real-time PCR, ELISA, expression of osteoblast markers in bone, and bone strength testing methods have been provided in the Supplementary Information.

### Generation of a d-galactose–induced aging model in mice

Mice were administered subcutaneous injections of D-gal at a dose of 100 mg/kg daily for 10 weeks to create a model of D-gal–induced aging. Saline was administered subcutaneously into control mice in place of D-gal. When D-gal treatment ended, the test agent IL-33 (100 ng/mouse/d) was intraperitoneally injected for 15 days. The Morris water maze test, NOR Novel Object Recognition test, and open field test were used to detect memory-related behavioral responses in mice with D-gal–induced aging to assess the impact of IL-33 on memory impairment.^13^^,^^14^

### In vivo studies

Male C57BL/6 J mice (weight, 22–25 g; age, 20 weeks) were used for in vivo studies. The study was conducted in accordance with current legislation on animal experiments and was approved by the Institutional Ethics Committee, Central Drug Research Institute (CPSCEA registration no. 34/1999, dated November 3, 1999, extended to 2015; approval reference no. IAEC/2021/83/renew 0; dated September 13, 2021). All of the mice were housed at 25°C with 12-hour light and 12-hour dark cycles. A normal feed pellet diet and water were provided ad libitum.

At the completion of the study, an autopsy of the mice was performed and bones were dissected followed by flushing out the bone marrow (BM). Using the Hisep LSM 1084 (Himedia) with a density (1.073 ± 0.0010 g/mL) gradient centrifugation method, total lymphocytes from the BM were separated. For the micro-CT (μCT) study, long bones were preserved in 70% isopropanol. Using an Imag cell separation magnet and CD4 Imag particles from BD Biosciences in accordance with the manufacturer’s procedure, pure CD4+ cells were recovered from the BM by positive selection. Following their purification, these cells were collected in Trizol for real-time PCR (qPCR). Serum samples were taken for ELISA.

### Biochemical estimation of neurodegenerative parameters

After the completion of neurobehavioral studies, the mice were killed and the frontal cortex and hippocampal brain regions were removed. To completely disrupt the cells, brain tissue was homogenized in ice-cold Tris-EDTA buffer (pH, -7.4; 0.1 M Tris–HCl; 1 mM EDTA; and 6 mM MgCl_2_). Brain oxidative stress markers comprising nitrite levels and lipid peroxidation in the form of malondialdehyde (MDA) levels were estimated in the tissue homogenate from both brain regions. Similarly, the antioxidant status in the form of reduced glutathione (GSH) levels was measured in both brain regions.^15^

### Protein extraction and Western blot analysis

Immunoblotting was performed to check the expression levels of osteogenic and senescent proteins after treatment of IL-33. For this purpose, whole-cell lysate was extracted from cultured mouse calvarial osteoblast (MCO) cells with cell lysis buffer comprising protease inhibitor cocktail and phosphatase inhibitor (Sigma, St. Louis, MO, USA). Protein concentration was estimated by BCA assay, and then separated on different percentages of SDS-PAGE, which were then electroblotted onto PVDF Polyvinylidene fluoride membranes (Immobilon-P; Millipore, Billerica, MA, USA). The membrane was then probed with Runx-2, type 1 collagen, p53, pRB, p21, PCNA, Ki-67, and β-actin antibodies as primary antibodies and incubated with secondary antibodies conjugated with horseradish peroxidase (HRP). On the Image Quant LAS 4000 (GE Healthcare) gel doc imaging system, immunodetection was performed with an enhanced chemiluminescence kit (Immobilon-P; Millipore, Billerica, MA, USA). ImageJ software was used for the quantification of blots.

Freshly extracted hippocampus and cortex (prefrontal cortex) regions were homogenized in cold RIPA radioimmunoprecipitation assay buffer lysis buffer with protease-phosphatase inhibitor cocktail (Sigma-Aldrich, St. Louis, MO, USA) to evaluate neurodegenerative parameters in rats. The BCA kit (Thermo Pierce, Rockford, IL, USA) was used to estimate the total protein content. A 40-μg sample of total protein was loaded, separated, and then transferred to a PVDF membrane (Millipore, Bedford, MA, USA). After blocking the PVDF membrane for 2 hours at room temperature with 5% bovine serum albumin (BSA), the membrane was incubated with primary antibodies for a whole night at 4°C. The primary antibodies used in this study were mouse anti–β-actin (1:2000; Millipore, USA), rabbit anti-p- tau(1:500; Santa Cruz Biotechnology, CA, USA), tau (1:500; Santa Cruz Biotechnology, CA, USA), mouse anti-BACE1 (1:1000; Millipore, USA), rabbit anti-p-CREB (1:1000; Millipore, USA), and rabbit anti-CREB (1:1000; Millipore, USA). The next day, the membranes were washed with TBST Tris-buffered saline with 0.1% Tween 20 detergent and left to incubate for 2 hours with the corresponding secondary antibodies (HRP conjugated anti-mouse IgG/anti-rabbit IgG; 1:5000; GeNei, India). A chemiluminescence substrate (ECL) Enhanced chemiluminescence from Millipore (MA, USA) was used to detect the band intensity, and image analysis software (Thermo Scientific, USA) was used to quantify the signal intensity.

### Flow cytometry

#### Intracellular staining of IL-17 and Treg staining

Intracellular staining for IL-17, IL-33, and Foxp3 (a Treg transcription factor) was carried out in accordance with the manufacturer’s instructions. We purchased the Treg staining kit from Biolegend (San Diego, CA). PBMCs were collected from BM and treated for 6 hours with 10 ng/mL phorbol 12-myristate 13-acetate, 250 ng/mL ionomycin, and 10 g/mL brefeldin A in order to stain IL-17A. Leucoperm reagents A and B (the fixation and permeabilization reagents) were then used to harvest, fix, and permeabilize the cells. IL-17 staining on ice with anti-mouse CD4, IL-17, and IL-33 antibodies (PE conjugated anti-mouse IL-17, APC conjugated anti-mouse CD4, and BV421A conjugated anti-mouse IL-33) was used to assess the percentage of CD4^+^ IL-17^+^ and CD4^+^ IL-33^+^ cells. The specificity of immunostaining was ascertained by background fluorescence of the cells incubated by isotype control of IgG. After intracellular staining, cells were washed twice with PBS and transferred in FACS tubes for analysis. FACS Aria (BD Biosciences Mississauga, ON, Canada) were used to quantify the percentage of IL-17^+^ CD4^+^ and IL-33^+^ CD4^+^ cells in all groups. Additionally, using BD Biosciences’ FACS Aria, the percentage of CD4^+ ^CD25^+^ and CD25^+^ FOXP3^+^ cells was determined.^2^

#### Isolation of CD4^+^ T cells to check gene expression

Using Hisep (Himedia, Navi Mumbai, India), BM cells were flushed out of the BM and PBMCs were extracted. The BD IMagnet Cell Separation Magnet was used to isolate CD4^+^ T cells through positive selection. Each step was carried out according to the manufacturer’s instructions. The cells were collected for RNA isolation after isolation and collected in 1 mL of TRIzol (Invitrogen). One microgram of total RNA was converted into cDNA using a cDNA synthesis kit. SYBR green chemistry was used to quantify gene expression for ROR-γt, STAT-3, TNF-α, Foxp3, and GAPDH, using an optimized method.

#### Micro-CT in long bones

Micro-CT of femur bones was performed using the Sky Scan 1076 CT scanner (Aartselaar, Antwerp, Belgium) following established procedures. With an X-ray source calibrated at 70 kV and 100 mA, trabecular areas within the bone samples were scanned at a nominal resolution of 18 mm per pixel. Using 100 projections over a 180° angular range, image slices were created, and the Sky Scan Nrecon program was used to reconstruct the images. Reconstruction was made easier by a modified Feldkamp approach, which made it possible to create a distributed network of pictures. The structural properties and density distribution within the trabecular bone network were then revealed by computing a number of trabecular bone parameters, including fractional bone volume (BV/TV), trabecular number (Tb.N), trabecular separation (Tb.Sp), and trabecular thickness (Tb.Th).^16^

### Statistical analysis

All values are expressed as mean ± SEM. The Student’s *t* test and 1-way or 2-way ANOVA with Bonferroni post hoc test were used to calculate the mean significant difference between the experimental groups. The statistical analysis was carried out using GraphPad statistical analysis software, version 8.01 (San Diego, CA, USA). At *p* < .033, differences were deemed statistically significant.

## Results

### IL-33 ameliorates d-galactose–induced suppression of osteoblast differentiation and represses osteoblast senescence

**Figure 1:**
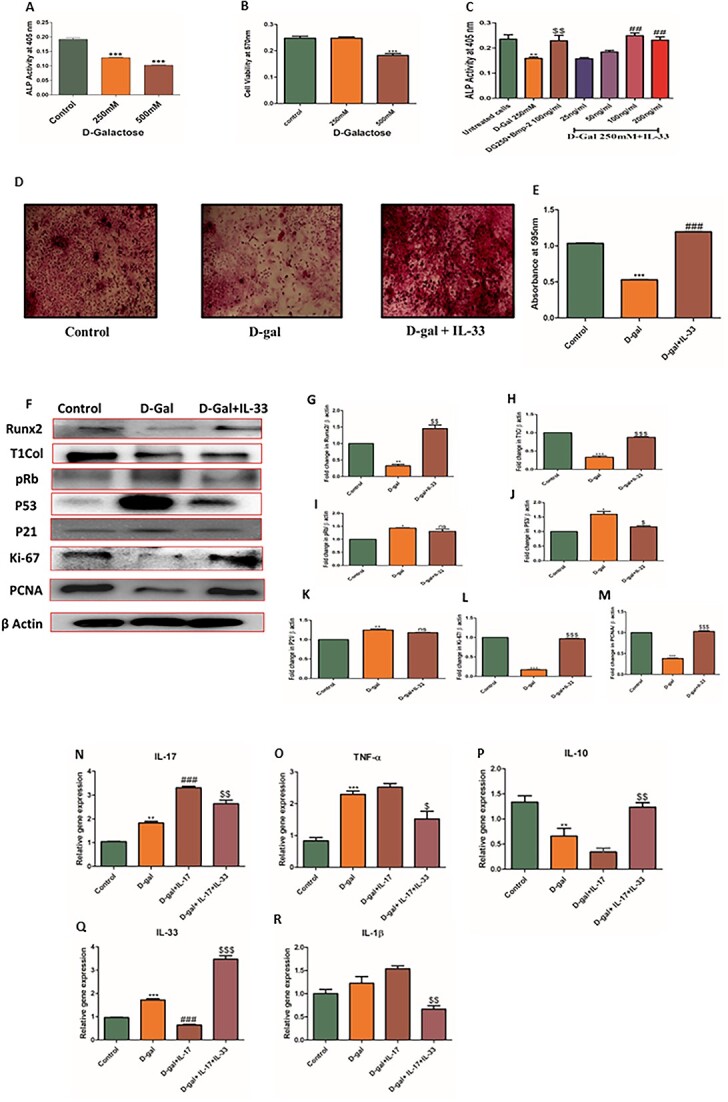
IL-33 ameliorates d-galactose (D-gal)–induced suppression of osteoblast differentiation, represses osteoblast senescence, and attenuates inflammatory responses in D-gal–stimulated osteoblasts by modulating expression of IL-17. (A) Alkaline phosphatase (ALP) activity was measured at 72 hours after D-gal administration to assess cell differentiation. (B) An MTT 3-(4,5-dimethylthiazolyl)-2,5-diphenyltetrazolium bromide test was used to determine cell viability at 72 hours after D-gal treatment. (C) ALP activity in the presence of IL-33 with positive control BMP-2. Data are expressed as mean ± SEM (*n* = 3). ^*^p < .05, ^**^p < .01, and ^***^p < .005 compared with control. ^$^*p* < .05, ^$$^*p* < .01, and ^$$$^*p* < .005 compared with D-gal. ^#^*p* < .05, ^##^*p* < .01, and ^###^*p* < .005 compared with D-gal + BMP-2. (D) Representative images of alizarin Red staining showing mineralization among different groups. (E) Quantification of mineralization using 10% CPC cetylpyridinium chloride. (F) Protein expression of Runx-2, type 1 collagen, P53, P21, pRB, PCNA, and Ki-67 gene in primary OB cells on treatment with D-gal and IL-33. (G–M) Quantification of protein expression using ImageJ software. (N–R) Relative mRNA expression of inflammatory cytokines such as IL-1β, TNF-α, and IL-17, IL-33, and IL-10 after 72 hours of treatment with D-gal, IL-17, and IL-33. Data expressed as mean ± SEM; *n* = 3. ^*^p < .05, ^**^p < .01, and ^***^p < .005 compared with control. ^$^*p* < .05, ^$$^*p* < .01, and ^$$$^*p* < .005 compared with D-gal.

As D-gal promotes cell senescence, alkaline phosphatase (ALP) activity, a characteristic of osteoblast formation, was initially measured in osteoblast cells pretreated with D-gal. d-Galactose was found to inhibit the ALP activity significantly at concentrations of 250 and 500 mM. Cell viability by MTT3-(4,5-dimethylthiazolyl)-2,5-diphenyltetrazolium bromide assay was checked at these concentrations. Cell viability was significantly less at 500 mM; however, at a 250-mM concentration the cells remained healthy. Hence, the concentration of 250 mM was chosen for further experiments (Figure 1A and B). Following this, the effect of IL-33 on osteoblast cell differentiation by ALP activity measurement was checked at concentrations ranging from 25 ng to 200 ng/mL in the presence of D-gal. It was found that IL-33 strongly promotes the differentiation of osteoblasts at 100- and 200-ng/mL concentrations, even in the presence of D-gal (Figure 1C). However, the effect was better at 100 ng/mL. BMP-2 was taken as a reference standard. Overall, this result demonstrated that D-gal–induced suppression of osteoblast differentiation was restored in the presence of IL-33. Additionally, mineral nodule formation was estimated among different groups, where we found less mineral nodule formation in D-gal–treated cells compared with control, while this effect was restored by IL-33 treatment as demonstrated by alizarin Red S staining (Figure 1D and E). Further, these observations were strengthened by evaluation of the expression of osteogenic Runx-2 and type 1 collagen in cells treated with IL-33 and D-gal. Osteogenic marker expression, like Runx-2 and type 1 collagen, was reduced in D-gal–treated osteoblasts; however, the expression was significantly increased in D-gal–induced osteoblasts treated with IL-33. Because D-gal promoted cell senescence, it was hence deemed reasonable to check the expression of senescence markers like p53, pRB, and p21. The protein levels of these markers were increased in D-gal–induced osteoblast cells. However, in the presence of IL-33, the expression levels of these senescent markers were significantly reduced. These results clearly show that D-gal–induced reduction in osteogenic markers was reversed by treatment with IL-33. On the other hand, D-gal led to an increase in osteoblast senescence, an effect mitigated by IL-33. We also checked proliferation markers like Ki-67 and PCNA to strengthen our findings; proliferation marker expression was reduced in D-gal–treated cells while these levels were significantly increased after treatment with IL-33 (Figure 1F–M).

### IL-33 attenuates inflammatory responses in d-galactose–stimulated osteoblasts by modulating expression of IL-17

Both IL-33 and IL-17 have been shown to act antagonistically in psoriatic inflammation, where IL-33 alleviates psoriatic inflammation by suppressing Th17 response.^17^ Hence, D-gal–treated osteoblast cells were incubated with IL-17, IL-17+IL-33, and levels of proinflammatory cytokines like TNF-α, IL-17, and IL-1β were determined. Expression of anti-inflammatory cytokine IL-10 was also determined. d-Galactose significantly elevated the expression of proinflammatory cytokines like IL-1-β, TNF-α, and IL-17, while the expression of anti-inflammatory cytokine like IL-10 was found to be reduced. IL-17 treatment was found to further intensify this effect. Interestingly, in IL-17–treated cells supplemented with IL-33, the levels of proinflammatory cytokines IL-1β, TNF-α, and IL-17 were suppressed and IL-10 expression was increased (Figure 1N–R). Overall, this shows that IL-17–mediated induction of proinflammatory cytokines was mitigated in the presence of IL-33.

### IL-33 treatment reduces formation of oxidative stress–related indices in d-galactose–induced mice

After the protective effects of IL-33 in D-gal–induced aging osteoblasts were ascertained, it was important to validate these results in suitable animal model. A mouse model of D-gal–induced aging was developed. To confirm that aging was induced, an evaluation of neurodegeneration-related oxidative stress parameters was performed.

**Figure 2:**
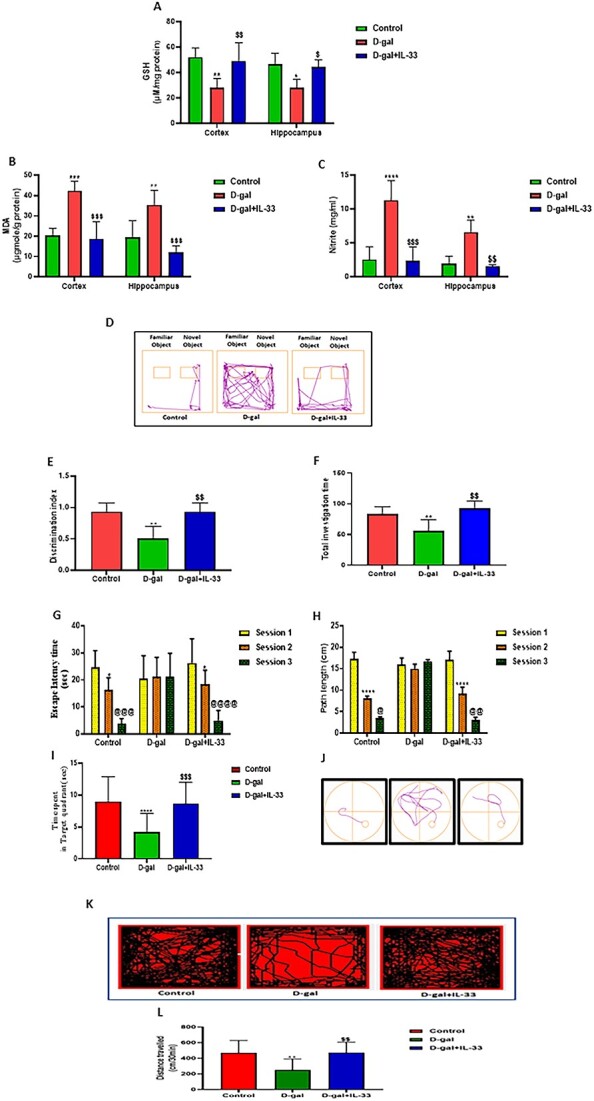
IL-33 treatment reduces oxidative stress–related indices, restores recognition memory, recovers spontaneous locomotor activity (SLA), and enhances spatial learning and memory retention in d-galactose (D-gal)–administered mice. (A) The bar graph represents GSH content in cortex and hippocampus regions. (B) The bar graph represents malondialdehyde (MDA) levels in cortex and hippocampus regions. (C) The bar graph shows total nitrite levels in cortex and hippocampus brain regions. (A–C) Data are expressed as mean ± SEM of *n* = 4 mice/group. Data were analyzed by 2-way ANOVA, followed by Bonferroni post hoc test. ^*^p < .0332, ^**^p < .0021, ^***^p < .0002, ^****^p < .0001; ^$^*p<*.0332, ^$$^*p<*.0021, ^$$$^*p<*.0002, ^$$$$^*P<*.0001. ^*^Control vs D-gal; D-gal vs IL-33. (D) Representative track plot of the path traced by animals from each of the groups. (E) The bar graph shows the discrimination index. (F) The bar graph represents the investigation time for novel and familiar objects by a mouse from each group. A Morris water maze test was used to assess spatial learning and memory functions. (G) The bar graph show latency termed as escape latency (in seconds) to reach the hidden platform. (H) The bar graph shows the mean path length (in centimeters). (I) A probe trial was performed to evaluate the time spent by mice in the target quadrant. (J) Representative track plot of the path traced by a mouse from each group. Exploratory behavior was assessed by recording SLA in terms of distance travelled in centimeters. (K) The representative image shows a track plot of the mice for 30 minutes in an open-field arena. (L) The bar graph shows the horizontal distance travelled over 30 minutes in an open-field arena. (D–L) All data are expressed as mean ± SEM of *n* = 6–8 mice/group. Data were analyzed by 1-way ANOVA followed by Bonferroni post hoc test. ^*^p < .0332, ^**^p < .0021, ^***^p < .0002, ^****^p < .0001; ^$^*p <* .0332, ^$$^*p <* .0021, ^$$$^*p <* .0002, ^$$$$^*p <* .0001. ^*^Control vs D-gal; D-gal vs IL-33.

We began our study by measuring the amount of MDA and nitrite generation to investigate the impact of IL-33 on oxidative stress burden in the cortex and hippocampal brain regions of mice. Further, to investigate the neuroprotective effect of IL-33 on the antioxidant defense system, we measured the GSH content (most significant antioxidants in the brain) in the same brain parts. GSH content was significantly lowered in the cortex (Figure 2A) and hippocampal regions (Figure 2A) of D-gal–induced aged mice as compared with control mice. IL-33 treatment in D-gal–induced aged mice resulted in a remarkable increase in GSH levels in the cortex and hippocampus regions (Figure 2A) when compared with D-gal–induced aged mice, suggesting the antioxidant effect of IL-33 against D-gal–induced neurotoxicity. We found that D-gal–induced aged mice displayed significantly increased MDA levels and nitrite content (Figure 2) in the cortex and hippocampus region, respectively, as compared with control mice. IL-33 treatment in D-gal–induced aged mice significantly reduced MDA levels and nitrite content (Figure 2B and C) in the cortex and hippocampus regions when compared with the D-gal–induced group.

### IL-33 resumes recognition memory, enhances spatial learning and memory retention, and recovers spontaneous locomotor activity in d-galactose–administered mice

Rodents exhibit a natural propensity to spend more time exploring unfamiliar (novel) objects than they do with familiar ones. This preference in investigating a novel object shows the use of learning and recognition memory. To check the effect of IL-33 treatment on recognition memory, we performed a novel object recognition test. Subcutaneous injection of D-gal decreased the discrimination index and total investigation time, whereas IL-33 treatment significantly increased the discrimination index and total investigation time (Figure 2D–F) in the D-gal aging model. Chronic D-gal administration impaired recognition memory in mice in contrast to control mice, which was resumed by IL-33 treatment.

To evaluate spatial learning and memory retention after IL-33 treatment in the D-gal–induced aging model, we conducted a Morris water maze experiment. Subcutaneous administration of D-gal did not result in any remarkable change in escape latency time in the second and third sessions as compared with the first session, in contrast to the control group, which indicates reduced spatial learning**.** Treatment with IL-33 significantly decreased the escape latency time in session 2 and session 3 and path length in the second and third sessions (Figure 2G and H) when compared with the first session, indicating reversal of spatial learning deficits. A significant decrease in time spent in the target quadrant was observed in the D-gal group in contrast to the control group, indicating a reduction in retention memory, which was resumed by IL-33 treatment (Figure 2I). A representative track plot of the path traced by the mouse in each group, followed during one of the trials in the third session (Figure 2J), shows that D-gal–administered mice took more time to reach the platform in contrast to the control group. Treatment with IL-33 significantly reduced this time duration, indicating an improvement in spatial memory.

IL-33 reversed D-gal–induced exploratory behavioral deficits in a mouse model of aging-like phenotypes. Assessment of spontaneous locomotor activity (distance travelled in centimeters) was performed for a period of 30 minutes in the Optovarimax apparatus. After D-gal treatment, locomotor activity (Figure 2K and L) showed a significant downward trend, while IL-33 treatment led to a significant increase in spontaneous locomotor activity (Figure 2K and L) as compared with D-gal–administered mice.

### Effect of IL-33 on senescence and osteogenic markers in d-galactose–induced aging in mice

**Figure 3:**
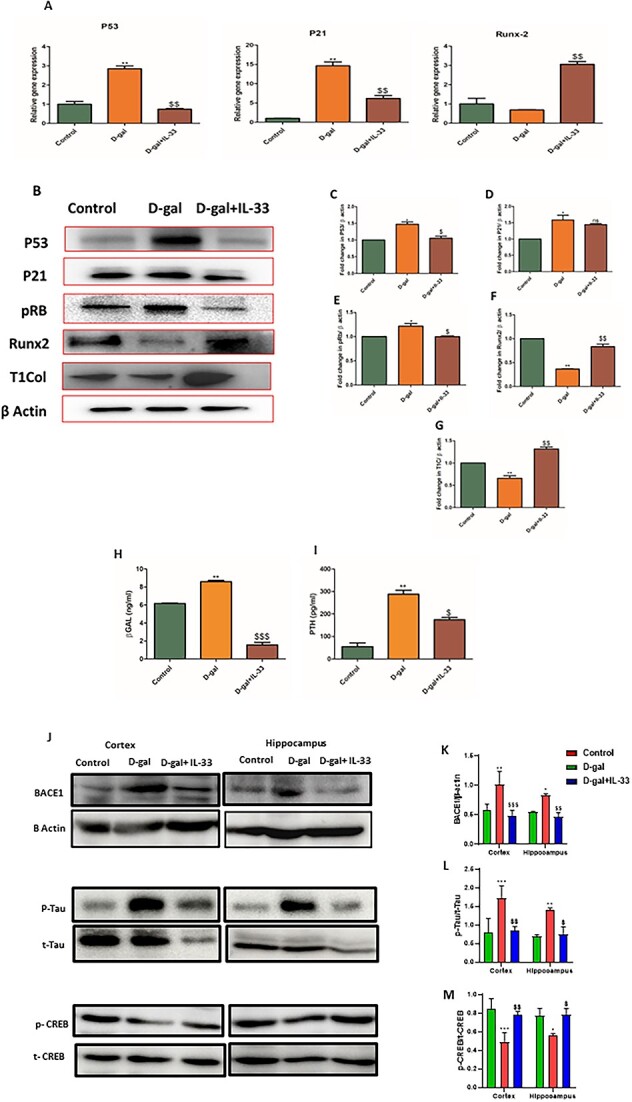
Effect of IL-33 on senescence and osteogenic and neurological markers. (A) Relative mRNA expression of senescence and osteogenic genes such as Runx-2, P53, and P21 in different groups. (B) Protein expression of Runx-2, type 1 collagen (T1Col), P53, P21, and pRB genes in different groups. (C–G) Quantification of protein expression using ImageJ software. (H) Serum beta-galactosidase (βGAL) levels. (I) Serum PTH levels. Data are expressed as mean ± SEM; *n* = 3. ^*^p < .05, ^**^p < .01, and ^***^p < .005 compared with control. ^$^*p* < .05, ^$$^*p* < .01, and ^$$$^*p* < .005 compared with d-galactose (D-gal). (J) Illustrative immunoblots show expression of BACE1, p-tau, tau, p-CREB, CREB, and β-actin in cortex and hippocampus regions. The bar graphs show quantification of relative protein density of (K) BACE-1, (L) p-tau, and (M) p-CREB in the cortex and hippocampus regions after normalization with β-actin, t-tau, and t-CREB, respectively. (K–M) Data are expressed as mean ± SEM of *n* = 3 mice/group. Data were analyzed by repeated-measures 2-way ANOVA and 1-way ANOVA, followed by Bonferroni post hoc test. ^*^P < .0332, ^**^p < .0021, ^***^p < .0002, ^****^p < .0001; ^$^*p <* .0332, ^$$^*p <* .0021, ^$$$^*p <* .0002, ^$$$$^*p <* .0001. ^*^Control vs D-gal; D-gal vs IL-33.

p53 modulates multiple downstream pathways, resulting in cellular senescence and other responses. Senescence indicators, among them p21, are identified as p53 target genes that cause cellular senescence. The pRB pathway is also a dominant effector of senescence. To evaluate the expression levels of these senescence markers in bone samples of D-gal and D-gal + IL-33 groups, RNA and protein lysates were prepared. The mRNA levels of p21 and p53 were elevated in the D-gal group (Figure 3A). However, treatment with IL-33 led to a reduction in the transcripts of these senescence markers (Figure 3A). A similar analysis was observed when the expression of these senescence markers was seen at the translational level, with an increase observed in p53, p21, and pRB in D-gal–treated groups, an effect that was corrected after IL-33 treatment (Figure 3B–G). Runx-2 mRNA expression was decreased in the D-gal group, while treatment with IL-33 restored the mRNA expression (Figure 3A). Runx-2 and type 1 collagen protein expression was decreased in D-gal–treated animals, while this was reversed in the D-gal group treated with IL-33. The results show the protective role of IL-33 in aging bone-loss conditions (Figure 3B–G). Serum PTH and beta-galactosidase levels are important markers with respect to aging bone-loss conditions. Beta-galactosidase is a key marker of cellular senescence and a high level of serum PTH indicates a hyperparathyroidism condition, which ultimately has adverse effects on bone and the nervous system. Therefore, it was important to check the levels of PTH and beta-galactosidase in D-gal–induced aging in mice and also in the presence of IL-33. We found that IL-33 treatment resulted in a decrease in serum PTH and beta-galactosidase levels, which was increased by D-gal treatment (Figure 3H and I).

### IL-33 treatment reduces BACE1 expression and tau pathology and regulates neuronal cell survival cues in a d-galactose–induced aging model

Proteins involved in the amyloidogenic pathway, which usually result in aggregation of Aβ-42 plaques, are considered as important hallmarks of aging. In an animal model, stopping BACE1 activity lowers the Aβ burden and improves cognitive function. As a result, blocking BACE1 activity may prevent 1 of the early pathogenic processes in Alzheimer’s disease. We found enhanced levels of BACE1 protein in cortex and hippocampus (Figure 3J and K) regions of D-gal–injected mice in contrast to control mice. IL-33 treatment in D-gal–administered mice significantly downregulated BACE1 protein levels in cortex (Figure 3J and K) and hippocampus (Figure 3J and K) regions, when compared with chronic D-gal–administered mice, suggesting that IL-33 has an inhibitory effect on the progression of amyloid pathology in D-gal–induced aged mice. In physiological conditions, tau protein plays an important role in the congregation and perpetuation of the structural stability of microtubule, but abnormally hyper-phosphorylated tau not only loses its biological activity and disassociates from microtubules, but also promotes its polymerization. Thus, soluble aberrant tau or its oligomers cause neuronal death and dementia. Tau phosphorylation at Ser 396 was remarkably enhanced in the cortex and hippocampus (Figure 3J and L) of D-gal–administered mice as compared with control mice. Treatment with IL-33 in D-gal–administered mice remarkably diminished the tau phosphorylation in cortex and hippocampus (Figure 3J and L) regions as compared with D-gal–administered mice.

CREB is a crucial component of the neuroprotective transcriptional network, and it is evident that CREB dysregulation is linked to a variety of neuropathological disorders. Since phosphorylated CREB binds to a particular sequence in the BDNF promoter and controls its transcription, it is 1 of the main regulators of neurotrophin responses. Thus, decreased p-CREB expression ultimately leads to downregulated expression of neurotrophins. Phosphorylated CREB levels have been reported to be diminished in brain regions of aged animals. D-gal administration significantly downregulated p-CREB in cortex and hippocampus (Figure 3J and M) regions as compared with the control group. Interestingly, IL-33 treatment in D-gal–induced aged mice significantly increased p-CREB levels in cortex and hippocampus (Figure 3J and M) regions as compared with D-gal–induced aged mice. Figure 3K-MFigure 5B–D shows the densitometric analysis of BACE1, p-tau, and p-CREB proteins.

### IL-33 protects against d-galactose–induced bone loss by improving bone microarchitecture, biomechanical strength, and maintenance of bone biochemical markers

**Figure 4:**
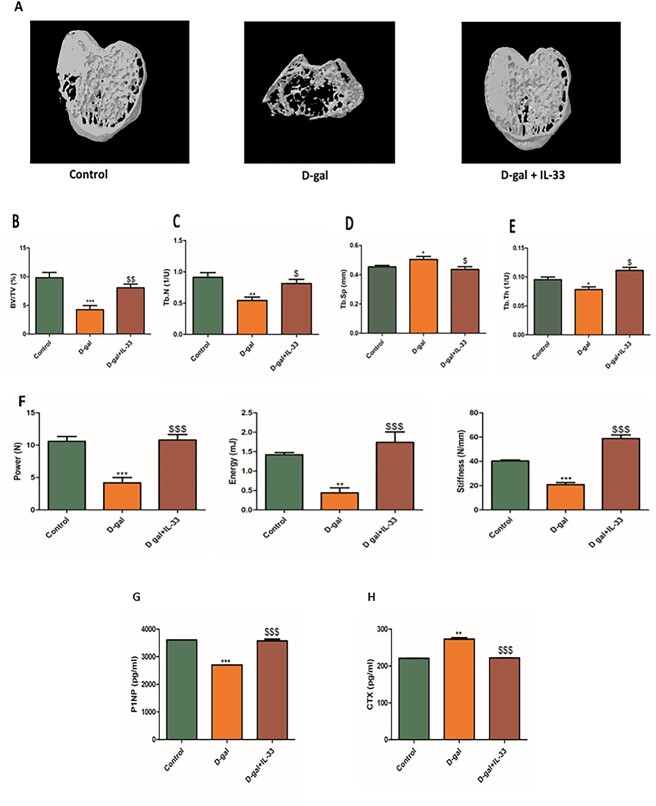
IL-33 protects against d-galactose (D-gal)–induced bone loss by improving bone microarchitecture and biomechanical strength. (A) Representative microCT images of femur bone after treatment with D-gal and D-gal + IL-33 (100 ng/mouse/d). (B) Bone volume/trabecular volume (BV/TV). (C) Trabecular number (Tb.N). (D) Trabecular separation (Tb.Sp). (E) Trabecular thickness (Tb.Th). (F) Bone strength parameters, such as energy, power, and stiffness of femur bone. (G) Serum P1NP levels. (H) Serum CTX levels. All values represent mean ± SE; *n* = 6. ^*^p < .05, ^**^p < .01, and ^***^p < .005 compared with control. ^$^*p* < .05, ^$$^*p* < .01, and ^$$$^*p* < .005 compared with D-gal.

The data were further strengthened by determining the role of IL-33 on trabecular bone microarchitecture and bone quality in aging-induced animals. For this, a study was carried out in male C57BL/6 J mice. The μCT scans and 3D reconstruction images revealed that bones treated with D-gal had severely damaged bone microarchitecture, with thin and less dense trabeculae. The D-gal–treated group had considerably decreased skeletal indices, such as BV/TV, Tb.N, and Tb.Th (Figure 4A–D). In contrast, treatment with IL-33 prevented substantial D-gal–induced bone loss by significantly boosting μCT parameters, such as BV/TV, Tb.N, and trabecular thickness Tb.Th (Figure 4A–D). Trabecular separation (Tb.Sp), which was elevated in D-gal–treated animals was restored to control levels in IL-33–treated animals Figure 4D). The data suggested that IL-33 at a dose of 100 ng/mouse per day could promote bone formation under aging bone-loss conditions. Furthermore, bone strength parameters showed that the D-gal group had lower bone strength than the control group, with lower energy, power, and stiffness. Treatment with IL-33 resulted in enhanced bone strength measurements, including a significant increase in energy, stiffness, and power (Figure 4F). Collectively, these data demonstrated that IL-33 restores the D-gal–induced deterioration in bone microarchitecture and improves the bone biomechanical properties*.* Additionally, the serum bone anabolic marker P1NP and resorption marker CTX were compared between the groups. Levels of P1NP were decreased in the D-gal group but returned to control levels in IL-33–treated mice (Figure 4G). On the contrary, the resorption marker CTX was high in D-gal–treated animals and its levels were decreased post–IL-33 treatment (Figure 4H). Taken together, these results reflect that senescence-induced changes in biochemical markers were corrected in animals treated with IL-33.

### Effects of IL-33 on Th17 cell proliferation and Treg cell and Th17/Treg balance

**Figure 5:**
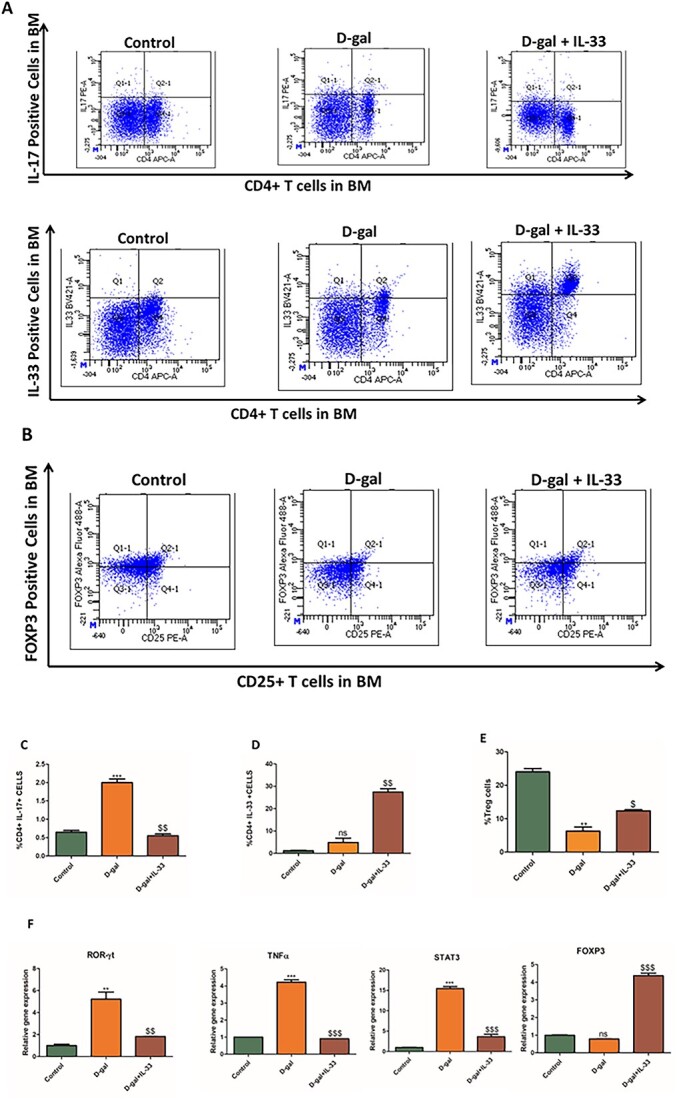
Effects of IL-33 on Th-17 cell proliferation and Treg cells. (A) FACS was used to detect the percentage of IL-17– and IL-33–positive cells in bone marrow (BM) PBMCs across all groups. (B) The percentages of Treg cells were detected in PBMCs isolated from different groups. (C–E) Quantitative representation of FACS data. *n* = 8 mice/group. Data are presented as mean ± SE. (F) mRNA transcript levels of ROR-γt, TNF-α, STAT-3, and FoxP3. Data are expressed as mean ± SEM; *n* = 3. ^*^p < .05, ^**^p < .01, and ^***^p < .005 compared with control. ^$^*p* < .05, ^$$^*p* < .01, and ^$$$^*p* < .005 compared with d-galactose (D-gal).

Numerous studies have shown the inverse relationship between IL-17 and IL-33.^6^ For this, we determined the role of IL-33 on Th17 differentiation in aged mice. FACS analysis of PBMCs from different groups indicates that D-gal mice produce more IL-17A^+^ cells than the control group. Interestingly, treatment with IL-33 restores IL-17A^+^ cells (Figure 5A and C). We also checked levels of IL-33 in D-gal–treated animals and found that the percentage of IL-33 was much higher in the treatment group. This might be an initial defense mechanism to counteract the increase in Th17 proliferation (Figure 5A and D). The proliferation of Treg cells, which maintain peripheral tolerance, was lower in the D-gal–treated group, while a significant enhancement was observed in IL-33–treated animals, albeit lower than in the control group (Figure 5B and E). Thus, IL-33 suppresses the proinflammatory Th17 proliferation and enhances Treg cells and thus protects the cell from aging-induced cellular senescence and bone loss. The key transcription factor for Th17 is ROR-γt and differentiation of Th17 cells is triggered by signal transducer and activator of transcription 3 (STAT-3); hence, the mRNA expression of both these genes was checked in CD4^+^ T cells isolated from different groups. Both ROR-γt and STAT-3 transcripts were elevated in the D-gal–treated group, while this phenomenon was reversed in the IL-33 group (Figure 5F). Foxp3, which is the critical transcription factor for Treg differentiation was decreased in the D-gal group, while mRNA expression was enhanced after IL-33 treatment (Figure 5F). TNF-α, which is a proinflammatory cytokine, was elevated in the D-gal group, an effect that was reversed after IL-33 treatment (Figure 5F). Overall, an increase in the proinflammatory and oxidative stress environment is created post–D-gal treatment, which enhances the aging process. This effect is ameliorated by IL-33 treatment.

## Discussion

The Th2 cytokine IL-33 is primarily produced by stromal cells in response to proinflammatory stimuli. It performs the roles of an alarmin, a conventional cytokine, and a nuclear factor that can regulate gene transcription. IL-33, an alarmin, is released upon cell damage and following cell death. It takes part in stress reactions and causes the production of more cytokines. It appears from clinical and experimental data that the IL-33/IL-31 axis plays a significant part in osteoporosis. In order to stop bone loss, IL-33 triggers Th2 cells to secrete IL-31 and prevents RANKL-dependent osteoclastogenesis.^18^ Despite these reports, the role of IL-33 and the mechanism by which it regulates skeletal biology is largely unexplored. IL-33 has also been implicated in cognitive impairment and dementia.^19^ As there are no reports of IL-33 in aging bone-loss conditions, these background data deemed it reasonable to study the role of IL-33 in aging conditions as patients with dementia conditions like Alzheimer’s are at risk of osteoporosis.^20^ In the current study, we explored the osteo- and neuroprotective effects of IL-33 in a model of D-gal–aging-induced mice. Chronic administration of D-gal causes deterioration of cognitive and motor skills, similar to symptoms of aging. It is therefore considered to be a suitable model of accelerated aging.^21^

We first began our studies by assessing the effect of D-gal on calvarial osteoblast cells and observed a decrease in ALP activity in D-gal–treated cells and chose 250 mM as the optimal concentration. Administration of IL-33 in D-gal–treated osteoblasts reversed the inhibition of ALP activity. IL-33 also led to an increase in osteogenic markers and a decrease in senescence markers like p53, p21, and pRB, indicating that senescence-inducing effects of D-gal are mitigated by IL-33. Senescent cells have high levels of proinflammatory cytokines like IL-17, TNF-α, and IL-1β and lower levels of anti-inflammatory cytokines like IL-10.^22^ A similar pattern was seen in D-gal–treated cells. IL-33 is known to attenuate IL-17 expression in EAE Experimental autoimmune encephalomyelitis and so D-gal–exposed osteoblasts were treated with IL-17 alone and with IL-33. IL-17 exaggerated inflammation, which was decreased in cells in which IL-33 was administered.^23^ This result also supported the immunoprotective potential of IL-33.

After confirming the osteogenic and anti-senescence effect of IL-33 in calvarial osteoblasts, the same was evaluated in a D-gal–administered mouse model. First, the neuroprotective and antioxidant effects of IL-33 were determined. d-Galactose–treated mice exhibited increased oxidative stress in cortical and hippocampal regions of the brain. These mice also exhibited dementia-like pathological characteristics in terms of elevated BACE1 and tau expression. In contrast, IL-33 treatment significantly diminished D-gal–induced memory loss and oxidative stress, which may be due to CREB pathway activation. Chronic subcutaneous administration of D-gal creates a phenotype in mice that closely mimics conditions of dementia. Accumulating evidence suggests that chronic subcutaneous injection of D-gal induces brain senescence in animal models, perhaps due to increased oxidative stress, inflammation, and apoptosis, together with lowering brain-derived neurotrophic factors.^24^ As per previous studies, we observed that D-gal administration potentially induced memory impairment and simultaneously induces oxidative stress parameters (MDA, nitrite), whereas it diminished concentrations of antioxidants (GSH). Oxidative stress and neuroinflammation are widely associated with various neurological disorders, such as anxiety, depression, and cognitive disorders, as well as linked to pathophysiology of neurodegenerative diseases.^25^ The generation of ROS and its role in defense must be optimally balanced for appropriate cellular function. A growing body of research indicates that low antioxidant levels in the brain cause synaptic loss and neuronal death.^26^ The nuclear transcription factor CREB controls neuronal survival, cognitive impairment, and creation of new memories.^27^ Reduced expression of CREB in rat hippocampal cultures as well as postmortem brain hippocampal tissues has been reported .^28^ Considering earlier research, we also found increased levels of oxidative stress and decreased levels of p-CREB and GSH in the cortex and hippocampus of aged mice, after D-gal administration, suggesting that chronic D-gal administration potentially induces oxidative stress and affects synaptic plasticity, which could be a factor in D-gal–mediated memory impairment. In contrast, IL-33 treatment remarkably decreased oxidative stress and increased GSH levels and p-CREB expression in the cortex and hippocampus of D-gal–administered mice, suggesting that IL-33 shows antioxidant capacity by reducing the level of MDA and nitrite. In the case of Alzheimer’s disease, the expression of the BACE1 enzyme has been found to be significantly elevated in cortex and hippocampus brain regions, thereby generating Aβ protein.^29^ In our study, we found increased tau phosphorylation and BACE1 levels after D-gal administration that was related to increased oxidative stress in cortex and hippocampus regions. In contrast, IL-33 treatment inhibited BACE1 activity and tau phosphorylation in cortex and hippocampus regions of the brain. In light of these evidences, rationally it can be proposed that IL-33 attenuates memory impairment through its intrinsic property to inhibit BACE-1 expression and tau phosphorylation. Our study suggests that IL-33 is a novel target for improving spatial and recognition memory-associated cognitive deficits in D-gal–administered mice. IL-33 may ameliorate D-gal–induced aging bone loss and memory impairment in mice by regulating IL-17, Tregs, BACE1 expression, and tau phosphorylation, thereby inhibiting inflammaging.

Among the key senescence inducers are included the p16^Ink4a^/Rb and p21^Cip1^/p53 pathways.^30^ Studies by Yu et al^31^ have shown that p53 plays a major role in the development of osteoporosis. Targeted clearance of p21-positive cells has been shown to prevent radiation-induced osteoporosis.^32^ The expression of these senescence markers was found to be elevated in D-gal–administered mice, while IL-33 treatment led to a decrease in p53, p21, and pRB expression. On the contrary, D-gal suppressed osteogenesis by inhibiting the expression of type 1 collagen and Runx-2, which is the master transcription factor for osteoblastogenesis. In senescent cells, both in vivo and in vitro, measuring β-galactosidase (β-GAL) activity at pH 6.0 is 1 of the most popular techniques for evaluating cellular senescence in vivo and in vitro.^33^ β-Galactosidase activity was found to be elevated in the D-gal group, while IL-33 administration led to a decrease in its activity. Thus, IL-33 truly exhibits anti-senescence effects. The data were further supported by detecting serum PTH, which is elevated in aging conditions like Alzheimer’s.^34^ Elevated serum PTH levels in the D-gal group were decreased in IL-33–treated animals.

PTH affects cognitive activities, which include a variety of dementia-related symptoms, and it can penetrate the blood–brain barrier through PTH receptors found throughout the brain. Furthermore, IL-17 is stimulated by PTH,^35^ the key regulator of bone remodeling; mechanistically, this leads to increased production of RANKL from osteoblasts and osteocytes, which contributes to robust osteoclastogenesis and ROS production, ultimately leading to neuroinflammation.

It is well known that trabecular bone loss in age-related osteoporosis results from decreased bone formation rather than increased bone resorption as in postmenopausal osteoporosis. A significant deterioration in trabecular bone network was observed as assessed by reduced bone tissue volume and trabecular number and thickness. Trabecular separation, which increases with aging, was also inflated in D-gal–treated animals. However, IL-33 was able to mitigate the deteriorating consequence of D-gal treatment. With aging, the bones become brittle and weak and are more prone to fractures. However, IL-33 improved the bone biomechanical properties, as evidenced by an enhancement in power, energy, and stiffness parameters, which were otherwise compromised after D-gal administration.

As aging is associated more with decreased bone formation, we determined the levels of the bone formation marker, serum P1NP, which was reduced in aged animals but elevated after IL-33 treatment. On the contrary, IL-33 decreased the bone resorption marker CTX, which was otherwise elevated in D-gal–administered animals. This finding indicates that IL-33 has a dual effect on bone cells, decreasing bone resorption by inhibiting osteoclast activity and promoting bone formation via osteoblasts, which confirmed its bone anabolic efficacy. Thus, IL-33 prevented aging-induced deterioration of bone microarchitecture, improved bone quality, and increased bone formation.

There is an increased frequency of Th17 cells in the aging population. We observed a similar pattern in which IL-17–positive cells where higher in D-gal–treated animals. On the contrary, a reduction in Foxp3-positive cells, important for Treg differentiation, was found in the D-gal group. IL-33 treatment reversed this effect by reducing IL-17–positive cells and increasing Treg cells. Also, mRNA levels of ROR-γt, a master transcription factor for Th17 and STAT3 that is an important factor for lineage commitment of Th17, were increased in D-gal–administered animals. These factors were suppressed after IL-33 treatment, which indicated that IL-33 might have a stimulatory role in aging bone by suppression of the Th17 cell population. FOXP3, a transcription factor for Treg cells, was elevated after IL-33 treatment, which was otherwise low in the D-gal–administered group and Tregs have been shown to reduce bone loss and osteoporosis. Thus, altogether, these studies support the neuroprotective and osteoprotective role of IL-33 in aging bone-loss conditions. Based on our studies, we propose that IL-33 may be an effective therapy in treating aging-induced dementia and osteoporosis.

## Supplementary Material

supplimentary_ziae101

## Data Availability

The data supporting the findings of this investigation are accessible from the corresponding author upon reasonable request.
