# Supplementary material for: IL-33 prevents age-related bone loss and memory impairment by suppression of Th17 response: evidence in a d-galactose–induced aging mouse model
Source: JBMR Plus. 2024 Aug 2;8(10):ziae101. doi: 10.1093/jbmrpl/ziae101 (PMC11365962; doi:10.1093/jbmrpl/ziae101)
Supplement: supplimentary_ziae101 [file supplimentary_ziae101.docx]

**Supplementary Information**

**IL-33 prevents age-related bone loss and memory impairment by suppression of Th17 response: Evidence in D-galactose induced aging model**

Saurabh Kumar Kaushal^1,2^, Parul^3^, Alok Tripathi^1,2^, Devendra Pratap Singh^1,2^, Ankita Paul^1^, Kumari Alka^3^, Shubha Shukla^2,3^, Divya Singh^1,2^

CSIR-Central Drug Research Institute, Sector10, Jankipuram extension, Sitapur road,

Lucknow, 226031

Email: [divya_singh@cdri.res.in](mailto:divya_singh@cdri.res.in)

^1^Division of Endocrinology, CSIR‐Central Drug Research Institute, Lucknow, Uttar Pradesh, India

^2^Academy of Scientific and Innovative Research (AcSIR), Ghaziabad, Uttar Pradesh, India

^3^Division of Neuroscience and Ageing Biology, CSIR‐Central Drug Research Institute, Lucknow, Uttar Pradesh, India

Correspondence Divya Singh Division of Endocrinology, CSIR‐Central Drug Research Institute, Lucknow, Uttar Pradesh, India

**Funding Information**

This study was funded by the Department of Science and Technology under the project GAP303, Council of Scientific and Industrial Research under the projects MLP-2028 and MLP- 2035, New Delhi.

**Mouse calvarial osteoblast (MCO) culture**

Applying standard protocols, calvarial osteoblasts were extracted from cell cultures taken from newborn mouse pups that were 1-2 days old. Following their surgical excision, the calvariae were treated to five separate digestions at 37°C using a solution containing 0.1% collagenase P and 0.1% dispase (Sigma, St. Louis, MO, USA). After being collected, centrifuged, and resuspended, cells released during the second to fifth digestions were seeded into T-25 cm2 flasks containing osteoblast growth medium, which included 10% foetal bovine serum (FBS, Gibco, Life Technologies, USA), 1% penicillin/streptomycin, and alpha MEM (Sigma, St. Louis, MO, USA).^(1)^

**Alkaline phosphatase assay**

After the trypsinization of MCO at 70–80% confluency and the seeding of 3000 cells/well in 96-well plates containing osteoblast growth media, ALP activity was examined. Following that, cells were treated with D-gal and D-gal + IL-33 using differentiation media that contained 50 µg/ml ascorbic acid and 10 mM beta-glycerophosphate (Sigma, St. Louis, MO, USA). Using Spectra MaxM2 and p-nitrophenylphosphate as a substrate, absorbance at 405 nm was used to measure total ALP activity (PNPP, Sigma, St. Louis, MO, USA).^(2)^

**Evaluation of cell viability**

Osteoblast cells were treated with D-gal (250 mM) for 72 hours in order to test the vibality of the cells. After that, the media from each well was aspirated and 20 μl of MTT reagent (5 mg/ml) was added, allowing the cells to incubate for three to four hours. After the incubation period, 100 μl of dimethyl sulfoxide (DMSO) was added to each well to dissolve the formazan crystals that are generated by metabolically active cells. Spectra MaxM2 was then used to detect absorbance at 570 nm.^(3)^

**Mineralization assay**

For mineralization experiments, osteoblasts were cultured in accordance with our previously published lab protocol. Cells were seeded in α-MEM medium, containing 10% foetal bovine serum, 50 mg/ml ascorbic acid, and 10 mM β-glycerophosphate. Cells were incubated for 14 days at 37°C in a humidified CO2 incubator. After completion of time, the cells were fixed with 4% para formaldehyde (PFA) for 30 minutes at room temperature, stained with 40 mM alizarin red S, which stains nascent calcium, and measured with a 10% cetylpyridinium chloride (CPC) solution. The absorbance was measured at 595 nm.^(4)^

.

**GSH measurement**

0.1 M sodium phosphate buffer (pH 8.0) and 6 mM 5, 5 dithiobis-2-nitrobenzoic acid (DTNB) were used to measured GSH. A yellow color product is formed when the reduction of DTNB takes place in the presence of reduced glutathione. The reaction mixture containing supernatant of tissue homogenate alongwith DTNB and sodium phosphate buffer, was incubated at room temperature for 10 minutes. An ELISA plate reader (BioTek, USA) was used to measure the reaction optical density (OD) at 412 nm. To determine the GSH concentration, reduced glutathione standard curve was prepared, and the result has been represented as micrograms per milligram of protein.

**Nitrite estimation**

Griess reagent was used to estimate the total nitrite level in brain tissue. In brief, tissue homogenate was centrifuged at 1600xg for 10 min and 100 μl of supernatant was incubated in a 96-well plate (except in blank wells) with 100 μl of Griess reagent at room temperature for 20 minutes. Fresh Griess reagent was prepared by mixing 1% sulfanilamide in 5% phosphoric acid with 0.1% N-1-napthyl-ethylenediamine in water. The optical density was determined with an ELISA plate reader (BioTek, USA) set at 540 nm. Nitrite standard curve was created to calculate the concentration of nitrite in tissue samples. The amount of nitrite has been expressed as micrograms per milligram of protein.

**Lipid peroxidation**

The amount of lipid peroxidation was determined by measuring malondialdehyde (MDA) in 0.5 N NaOH with 30% trichloroacetic acid (TCA), 5 N HCl, and 2% thiobarbituric acid (TBA). TBA was added after the tissue homogenate had been mixed with TCA and HCl. The reaction mixture was centrifuged at 12,000 × g for 10 minutes, after being boiled for 15 minutes at 90 °C. The supernatant was collected and the intensity of pink color was quantified at 532 nm, using an ELISA plate reader (BioTek, USA). A tetraethoxypropane (TEP) standard curve was prepared to calculate the concentration of MDA, which is represented as nanomoles per milligram of protein.

**Behavioral tests**

**Novel Object Recognition test**

Novel object recognition test was performed to assess the working memory after IL-33 treatment by following our previously published protocol with minor modifications.^(5)^ The test was completed in three consecutive days that included habituation day, training day and testing day. On habituation day, each mice was habituated for 10 minutes in arena without placing any object. On the training day, each mice was allowed to investigate two similar objects for 10 minutes. Next on the testing day, one of the training object was replaced a with novel object. In normal condition mice shows innate preference for novel object, thus time spend with familiar and novel object was recorded using Anymaze software for each mice of all the groups. For estimating, the difference among all the groups, percent of total investigation time and discrimination index was calculated.


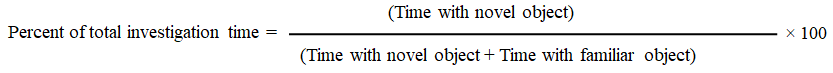


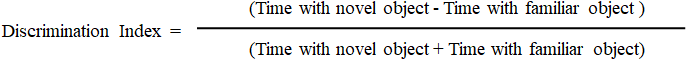


**Morris water maze test**

To assess the spatial learning and memory after IL-33 treatment in D-gal administered mice, Morris water maze test was performed in all the groups of experimental mice by following our previously published protocols with minor modifications.^(6)^ Test was conducted in a round pool (122 cm × 51 cm) with non- reflective interior surface, having a platform (10 cm diameter) hidden 1-2 cm below the water surface, at optimum temp of 25 ± 1^0^C. The whole experiment was completed in 3 sessions (days), each session consisting of three trials and all for 2 minutes’ time duration, followed by probe trial on day 4 of experiment. From session 1 to 3, mice were trained to memorize the platform location in the water filled tank for this, mice were allowed to swim in water tank for 2 minutes and each trial culminated when either the mice arrived on the platform or time period of 2 minutes is completed. For acquisition of memory, we calculated latency to reach the platform for all the three sessions. While in probe trial, (day 4) platform was pulled out and animal was allowed to swim in water tank for 1 minute. Complete experiment was recorded by using camera placed over the water tank and associated with Any maze software.

**Open field test**

We performed open field activity test activity according to our previously published protocol with minor modifications.^(7)^ To assess locomotor activity, rats were tested in an open-field arena of Optovarimax (Panlab, Harvard Apparatus, USA). Experimental arena was made up of transparent plexiglass boxes (42.5 cm x 42.5 cm) with a grid of completely automated infrared emitters and detectors. The number of infrared beams traversed by mice during horizontal walking, provides the data. Prior to the test phase, initially mice were acclimatized in apparatus for 10 minutes and after acclimatization, locomotor activity of each mouse was recorded for 30 minutes. After completion of experiment for each mouse, apparatus was swabbed with 10% ethyl alcohol to avoid the odor interference for next mice. Data were recorded by Acitrack software during the entire experiment.

**Total RNA Isolation and Quantitative Real-Time-PCR**

Trizol (Invitrogen) was also used to extract total RNA from isolated CD4^+^ T cells in each of the in-vivo groups and also from different groups of cultured cells that is Control, D-gal treated cells, D-gal + IL-33 treated, D-gal + IL-17 treated and D-gal + IL-17 + IL-33 treated. Utilising cDNA synthesis kit, cDNA was generated from one microgram of total RNA. Gene expression for ROR-γt, STAT-3, TNF-α, Foxp3, various pro-inflammatory (TNF-α, IL-1β, & IL-17), anti-inflammatory (IL-10, IL-33), osteogenic (Runx-2, Type 1 col) and senescence-associated (p53, p21, p16, etc.) genes a house-keeping gene, GAPDH, were quantitatively determined using SYBR green chemistry in accordance with an optimised procedure.

**Table**

Sequences of RT-PCR primers.

| **S.NO.** | **Gene name** | **Primer sequence** |
| --- | --- | --- |
| **1** | GAPDH | F-AGCTTGTCATCAACGGGAAG  R-TTTGATGTTAGTGGGGTCTCG |
| **2** | TNF-α | F-TGCCTATGTCTCAGCCTCTTC  R-GAGGCCATTTGGGAACTTCT |
| **3** | IL-1β | F-TTGACGGACCCCAAAAGAT  R-GAAGCTGGATGCTCTCATCTG |
| **4** | IL-10 | F-ATTTGAATTCCCTGGGTGAGAAG  R-CACAGGGGAGAAATCGATGACA |
| **5** | IL-17 | F-CAGGGAGAGCTTCATCTGTGT  R-GCTGAGCTTTGAGGGATGAT |
| **6** | IL-33 | F-GTCTCCTGCCTCCCTGAGTA  R-CGAGACGTCACCCCTTTGAA |
| **7** | STAT-3 | F-GGAAATAACGGTGAAGGTGCT  R-CATGTCAAACGTGAGCGACT |
| **8** | ROR-γt | F-CACTGCCAGCTGTGTGCT  R-TGCAAGGGATCACTTCAATTT |
| **9** | RUNX-2 | F-CATGTTCAGCTTTGTGGACCT  R-GCAGCTGACTTCAGGGATGT |
| **10** | FOXP-3 | F-AGAAGCTGGGAGCTATGCAG  R-GCTACGATGCAGCAAGAGC |
| **11** | P21 | F-CACCGAGCCTGTTTCTCTGT  R-CCACAGCACAGGAGTCACAT |
| **12** | P53 | F-GTGCTCACCCTGGCTAAAGT  R-TGGGAAGGAGGAGGATGAGG |

**Measurement of bone-relevant serum parameters**

For biochemical parameters analysis, blood was collected from each animal group and centrifuged at 4,000 rpm for 30 minutes to separate the serum component. The serum levels of PTH (serum Mouse I-PTH (intact Parathormone), P1NP and CTX ELISA Kit (Elabscience Biotechnology Inc., Donghu Hi-Tec Development area, Wuhan, Hubei, China) and Mouse B-galactosidase, β-GAL ELISA Kit from BT Lab were determined using enzyme-linked immunosorbent assay (ELISA) kits according to manufacturer's protocols.

**Bone strength testing**

The bone strength was measured using a previously described methodology. Femur bones were initially prepared by removing muscles and ligaments, after then horizontally positioned on a bone strength tester machine (TK252C, Muromachi Kikai Co. Ltd., Tokyo, Japan) with a 1cm distance and a loading pin positioned at the femur's midshaft. The load-displacement curves acquired during the testing method were then analysed to establish the strength properties of the femur bone, such as power, energy, and stiffness.^(8)^

**Expression of osteoblast markers in bone**

Muscles were extracted from several animal types, and bone marrow was removed afterwards. Tibia and femur were cleaned, combined, and processed in liquid nitrogen. The frozen powder that resulted was then put in tubes containing Trizol and RIPA Lysis buffer to isolate total RNA and protein respectively. After this extraction, the bone samples were subjected to Western analysis and qPCR to evaluate protein levels and gene expression.

1. Kureel J, Dixit M, Tyagi AM, Mansoori MN, Srivastava K, Raghuvanshi A, Maurya R, Trivedi R, Goel A, Singh D. miR-542-3p suppresses osteoblast cell proliferation and differentiation, targets BMP-7 signaling and inhibits bone formation. Cell Death Dis. [Internet]. 2014 Feb 6;5(2):e1050. Available from: http://www.ncbi.nlm.nih.gov/pubmed/24503542

2. Ahmad N, Kushwaha P, Karvande A, Tripathi AK, Kothari P, Adhikary S, Khedgikar V, Mishra VK, Trivedi R. MicroRNA-672-5p Identified during Weaning Reverses Osteopenia and Sarcopenia in Ovariectomized Mice. Mol. Ther. Nucleic Acids [Internet]. 2019 Mar 1;14:536–49. Available from: http://www.ncbi.nlm.nih.gov/pubmed/30769134

3. Prakash R, Mishra T, Dev K, Sharma K, Kuldeep J, John AA, Tripathi A, Sharma C, Arya KR, Kumar B, Siddiqi MI, Tadigoppula N, Singh D. Phenanthrenoid Coelogin Isolated from Coelogyne cristata Exerts Osteoprotective Effect Through MAPK-Mitogen-Activated Protein Kinase Signaling Pathway. Calcif. Tissue Int. [Internet]. 2021 Jul;109(1):32–43. Available from: http://www.ncbi.nlm.nih.gov/pubmed/33675370

4. Tyagi AM, Gautam AK, Kumar A, Srivastava K, Bhargavan B, Trivedi R, Saravanan S, Yadav DK, Singh N, Pollet C, Brazier M, Mentaverri R, Maurya R, Chattopadhyay N, Goel A, Singh D. Medicarpin inhibits osteoclastogenesis and has nonestrogenic bone conserving effect in ovariectomized mice. Mol. Cell. Endocrinol. [Internet]. 2010 Aug 30;325(1–2):101–9. Available from: http://www.ncbi.nlm.nih.gov/pubmed/20570709

5. Leger M, Quiedeville A, Bouet V, Haelewyn B, Boulouard M, Schumann-Bard P, Freret T. Object recognition test in mice. Nat. Protoc. [Internet]. 2013 Dec;8(12):2531–7. Available from: http://www.ncbi.nlm.nih.gov/pubmed/24263092

6. Tiwari V, Mishra A, Singh S, Mishra SK, Sahu KK, Parul, Kulkarni MJ, Shukla R, Shukla S. Protriptyline improves spatial memory and reduces oxidative damage by regulating NFκB-BDNF/CREB signaling axis in streptozotocin-induced rat model of Alzheimer’s disease. Brain Res. [Internet]. 2021 Mar 1;1754:147261. Available from: http://www.ncbi.nlm.nih.gov/pubmed/33422534

7. Walsh RN, Cummins RA. The Open-Field Test: a critical review. Psychol. Bull. [Internet]. 1976 May;83(3):482–504. Available from: http://www.ncbi.nlm.nih.gov/pubmed/17582919

8. Rai R, Kumar S, Singh KB, Khanka S, Singh Y, Arya KR, Kanojiya S, Maurya R, Singh D. Extract and fraction of Musa paradisiaca flower have osteogenic effect and prevent ovariectomy induced osteopenia. Phytomedicine [Internet]. 2021 Dec;93:153750. Available from: http://www.ncbi.nlm.nih.gov/pubmed/34662767
